# Calciphylaxis: A Rare Complication With a Fatal Outcome in End-Stage Renal Disease

**DOI:** 10.7759/cureus.45557

**Published:** 2023-09-19

**Authors:** Ahmed D Khudair, Aiman D Khudair, Mohamed Awadh, Nuria S Pérez Romano, Abdulla Darwish

**Affiliations:** 1 Department of Pathology and Laboratory Medicine, Royal College of Surgeons in Ireland - Bahrain, Muharraq, BHR; 2 Department of Pathology and Laboratory Medicine, Bahrain Defense Force Hospital - Royal Medical Services, Riffa, BHR; 3 Department of Nephrology, Bahrain Defense Force Hospital - Royal Medical Services, Riffa, BHR

**Keywords:** warfarin-induced skin necrosis, end stage renal disease (esrd), skin necrosis, calcific uremic arteriolopathy, dialysis, warfarin, end-stage renal disease, calciphylaxis

## Abstract

Calciphylaxis, or calcific uremic arteriolopathy, is a rare and deadly disease that affects patients with end-stage renal disease (ESRD). It typically manifests in the abdomen and lower extremities. We present a case of a 59-year-old female patient on dialysis due to ESRD who complained of a three-week history of hemorrhagic and painful bilateral lower limb lesions. The predominant clinical suspicion was warfarin-induced skin necrosis (WISN); however, the persistence of unresolved skin lesions post-warfarin cessation generated the impression of calciphylaxis. A skin biopsy confirmed the classical histological findings associated with calciphylaxis. This paper highlights the possible importance of warfarin being an inciting event, as well as the early differentiation between the presentations of WISN and calciphylaxis.

## Introduction

Calciphylaxis, also known as calcific uremic arteriolopathy, is a rare disease that most commonly affects patients with end-stage renal disease (ESRD). The aforementioned is termed uremic calciphylaxis, but in the absence of ESRD, it can be referred to as non-uremic calciphylaxis [1-3]. Calciphylaxis is characterized by the obstruction of small vessels due to calcification in the subcutaneous fat and dermis, which results in extremely painful skin lesions [4,5]. Skin lesions may first manifest as plaques, nodules, and indurations, eventually evolving to ulcers with a stellate shape and black eschars [4,5]. It usually occurs in the abdomen and the lower extremities [5]. Calciphylaxis, which carries a high one-year mortality rate of over 50%, is often complicated by infection of the underlying skin lesion, leading to sepsis [6]. In addition to the poorly understood pathogenesis of calciphylaxis, there is a paucity of approved treatment regimens. The most commonly used treatments, sodium thiosulfate (STS), surgical parathyroidectomy, cinacalcet, hyperbaric oxygen therapy (HBOT), and bisphosphonates, are reported to have no significant clinical benefit in patients suffering from calciphylaxis concurrent with chronic kidney disease (CKD) [2,7,8]. In this report, we present a case of fatal calciphylaxis in a 59-year-old female with ESRD.

## Case presentation

A 59-year-old female presented to the dermatology clinic after a referral from the hemodialysis unit due to hemorrhagic, painful lesions over both legs. The patient’s medical history was significant for hypothyroidism, essential hypertension, hyperlipidemia, type 2 diabetes mellitus, and stage five CKD on hemodialysis. She had a body mass index of 40.74 kg/m^2^ and was a non-smoker with no known allergies. Eight years ago, coronary angiography was performed and revealed normal coronary arteries. She had been on regular hemodialysis for the previous four years.

Initially, the patient noticed subcutaneous thickening in both thighs. She was advised to stop warfarin, which she has been on for four months due to a previous open-heart surgery for a double valve replacement. The subsequent clinical impression was warfarin-induced skin necrosis (WISN). The patient's laboratory investigations are recorded in Table 1.

**Table 1:** Laboratory investigations of an end-stage renal disease patient with calciphylaxis at presentation and three months prior to presentation

Laboratory investigations	Reference ranges	Values three months prior	Values at initial presentation
White blood cell count (x10^9^/L)	4–11	7.37	5.5
Hemoglobin (g/dL)	13-18	9.6	8.4
Albumin (g/L)	35-52	44.7	33.5
Alkaline phosphatase (IU/L)	40-129	188	261
C-reactive protein (mg/L)	0-5	-	65
Phosphorus (mmol/L)	0.81-1.45	0.93	2.34
Parathyroid hormone (pg/ml)	16-65	169	229
Creatinine (µmol/L)	62-106	569	522
Urea (mmol/L)	2.76-8.07	19.4	16.4
Vitamin D (nmol/L)	30-200	66.7	57.1
Serum calcium (mmol/L)	2.15-2.5	2.55	2.41
Urea clearance (Kt/V)	≥1.2	<1.2	<1.2

**Figure 1:**
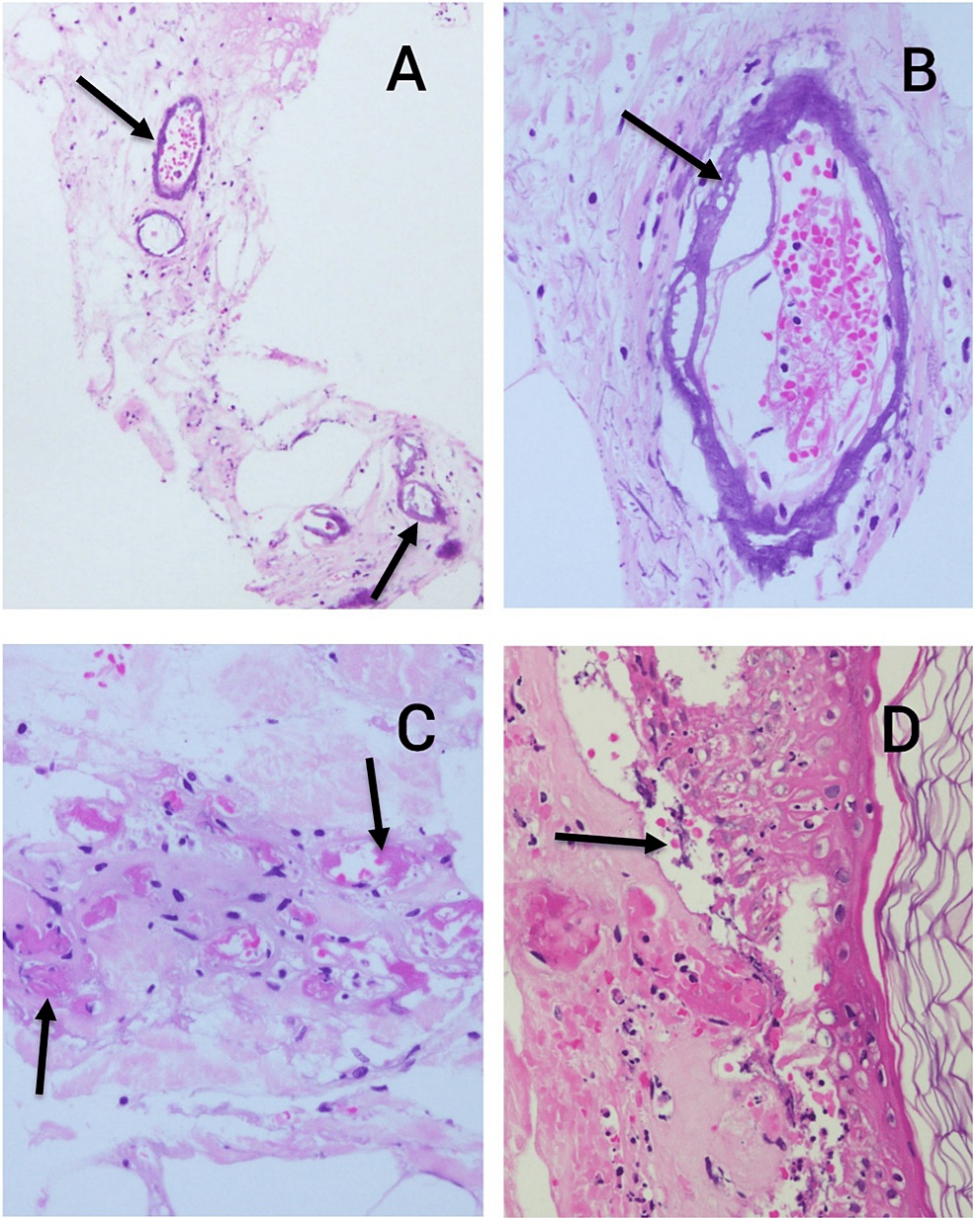
Hematoxylin and eosin images of calciphylaxis lesions identified in a patient with end-stage renal disease A: Multiple blood vessels with calcified walls in the subcutaneous tissue (arrows); B: High-power view of a blood vessel with a calcified wall; C: Blood vessels containing microthrombi (arrows); D: Skin showing separation and necrosis (arrow) with dermal fibrosis and thrombosed blood vessels.

A CT scan showed diffuse subcutaneous edema along the lateral aspect of the thigh with focal nodular fat thickening. There was no calcification or fluid collection reported. A mixed combination of beta-sitosterol and mupirocin ointment for the ulcers, erosions, and blisters was prescribed. Her skin lesions continued to deteriorate to painful induration, with erosions over both legs and a necrotic ulcer over her left leg where the skin biopsy was performed. On microscopic evaluation, the biopsy of the lower limb revealed fibro-adipose tissue demonstrating blood vessels with thick, heavily calcified walls, some of which contained thrombi (Figures 1A-1C).

The adjacent tissue showed reactive vascular proliferation with endothelial swelling, interstitial calcification, and focal fat necrosis. The overlying skin showed epidermal separation with early necrosis (Figure 1D). Overall, the appearances were consistent with calciphylaxis.

The ultrasound arterial and venous Doppler study was suboptimal due to the patient's diffuse soft tissue swelling over both legs; however, it ruled out deep vein thrombosis (DVT). Other significant findings included mild lumen narrowing with biphasic flow of the bilateral popliteal arteries, as well as mild to moderate lumen narrowing over the bilateral anterior tibial artery and posterior tibial artery.

A multidisciplinary approach to treatment was initiated and included wound care, pain management, empirical antibiotics, and a daily session of hemodialysis prescription-optimized with low calcium dialysate and 25mg of STS given intravenously post-hemodialysis three times per week. Hyperbaric oxygen therapy was recommended but was not performed due to the patient’s clinical condition. The color of the skin around the site of the blister turned black, culminating in dry gangrene. She was admitted to the medical ward due to septicemia secondary to calciphylaxis six weeks after the initial presentation. Laboratory tests showed an elevated level of procalcitonin of 2.7 µg/L (range: >2 indicates a high risk of systemic infection) and C-reactive protein of 330 mg/L (normal range: 0-5). Antibiotics were administered, and a surgical and dermatological review was sought for the skin wound. Microbiology culture of the skin wound grew *Acinetobacter baumannii*.

Her condition deteriorated, and she was shifted to the intensive care unit, where she required intubation, mechanical ventilation, and inotropic support. Antibiotic therapy with meropenem and vancomycin was administered according to the culture results and infectious disease specialists' recommendations. Continuous renal replacement therapy was started. Additionally, she developed ventilator-associated pneumonia and cardiac ventricular arrhythmias. The arrhythmias were defibrillated, and anti-arrhythmic medication was administered. Unfortunately, the patient's skin lesions extended from the lower limbs to the trunk and upper limbs. Eventually, the patient suffered cardiac arrest and succumbed to complications in one month.

## Discussion

Calciphylaxis, a rare but lethal condition, appears as painful skin plaques or subcutaneous nodules. These skin lesions adopt a mottled, stellate purple appearance. It can progress to necrotic eschars, non-healing ulcers, and eventually gangrene [9]. On histology, it is characterized by calcium deposition on the medial layer of vessel walls and hyperplasia or fibrosis of the intima, in addition to thrombosis of small vessels [10]. The risk factors for calciphylaxis that our patient possessed included female sex, diabetes mellitus, hypoalbuminemia, warfarin usage, hyperphosphatemia, obesity, and length of dialysis [5]. Differential diagnoses for a skin lesion appearing on a hemodialyzed patient include but are not limited to, calciphylaxis, WISN, heparin-induced thrombocytopenia, necrotizing fasciitis, cellulitis, and ecthyma [7].

The gold standard of diagnosis for calciphylaxis is a skin biopsy; however, due to its associated risk, it is controversial among the medical community as it may cause necrosis, infection, or facilitate the development of new lesions [4,5,10]. The diagnosis can also be supported by clinical findings of necrotic skin lesions with risk factors significant for calciphylaxis [10]. Our patient was on warfarin, and its use is associated with an increased risk for calciphylaxis [11]. The key player involved in its pathogenesis is matrix Gla protein (MGP), which plays a role in the inhibition of vessel wall calcification [11]. This protein requires a vitamin K-dependent enzyme to become carboxylated, which in turn leads to its ability to properly function. Since warfarin inhibits the carboxylation of vitamin K, it is reported that a 0.1 unit decrease in relative carboxylated MGP is associated with a two-fold increase in calciphylaxis risk [11]. This specific mechanism could potentially explain the underlying pathogenesis of warfarin-associated calciphylaxis (WAC).

Importantly, the recognition of calciphylaxis plays a major role in determining the mortality rate. Patients with non-ulcerating lesions, or early calciphylaxis, possess a mortality rate of 33%, whereas patients who have ulcers jump to a mortality rate of 80% [12]. This provides a justification for the importance of early recognition and subsequent management of calciphylaxis to potentially lower the mortality rate. The differentiating features between WISN and calciphylaxis are that the former appears due to recent warfarin initiation, while the latter is suspected in a patient with ESRD, hyperphosphatemia, and chronic warfarin usage presenting with painful plaques and ulcers in fatty tissue [13]. Another differential is WAC, which has an average onset of 32 months and presents with indurated net-like purpuric lesions and necrosis, most commonly on the lower extremities [14]. However, WISN presents with hemorrhagic bullae with retiform purpura three to 10 days post warfarin commencement [14]. The possibility of WAC occurring in our patient is unlikely given the timeframe, since our patient was on warfarin for only four months. However, in one case-control study, warfarin therapy showed a significant 10.1-fold increased risk for calciphylaxis [15].

Currently, there are no FDA-approved treatments indicated for calciphylaxis; however, the most popular and widely utilized medication is intravenous (IV) STS [5]. A recent meta-analysis showed no association between the improvement of calciphylactic skin lesions or survival with the usage of IV STS in patients suffering from CKD [16]. The mainstay of treatment, in addition to STS, is discontinuing all offending agents such as calcium supplements, warfarin, and steroids [17]. Furthermore, emphasis must be placed on pain relief, daily wound care, the adequacy of hemodialysis, and the correction of electrolyte abnormalities [17]. Additionally, the use of bisphosphonates, calcimimetics, vitamin K, HBOT, an infusion of a low-dose tissue plasminogen activator, or parathyroid surgery can also be considered. However, most of these treatment modalities are all derived from observational data, and there are no current approved regimens indicated for the treatment of calciphylaxis [8,17]. Furthermore, the five most common treatments showed no significant clinical benefit in CKD patients [8]. More randomized-controlled trials (RCTs) need to be conducted in order to decipher an optimal management plan for patients with calciphylaxis.

## Conclusions

Calciphylaxis continues to be a very rare and debilitating disease with a high mortality rate. The lack of approved treatment regimens, coupled with the underrecognized masquerading presentation, often leads to a very poor prognosis. Prompt recognition is crucial in the early stages of calciphylaxis since mortality rates skyrocket once lesions start to ulcerate. More RCTs and case reports are needed to better identify the early initial clinical image of calciphylaxis in order to promptly treat and cure this deadly disease.
